# Insecticide-treated nets (ITNs) in Africa 2000-2016: coverage, system efficiency and future needs for achieving international targets

**DOI:** 10.1186/1475-2875-13-S1-O29

**Published:** 2014-09-22

**Authors:** Samir Bhatt, Peter Gething

**Affiliations:** 1 University of Oxford, UK

## 

Insecticide-treated nets (ITNs), which comprise conventional (cITNs) and long-lasting insecticidal nets (LLINs), are the most widely used intervention for malaria control in Africa today. At least 700 million such nets have been financed and delivered to countries since the onset of the Roll Back Malaria (RBM) partnership at the turn of the millennium, but coverage remains inadequate. Better understanding of how and why these volumes of net deliveries translate into the levels of access and use seen in households across Africa could improve the monitoring of coverage indicators, highlight opportunities for systematic efficiency gains, and allow more accurate assessment of resources needed to meet international targets.

We used a simple compartmental model to represent the system linking ITN deliveries to countries, distribution to households, and resulting coverage at the national level. We assembled coverage data from 93 national surveys representing 861,000 households between 2000 and 2013 and triangulated these against yearly ITN manufacturer delivery data and national malaria control programme ITN distribution reports for 44 sub-Saharan countries. We fitted the model to these data using Bayesian inference with minimal prior assumptions.

We present contemporary estimates of LLIN coverage for all endemic African countries since the year 2000. We then explore the impact of two potential inefficiencies: uneven net distribution between households and rapid rates of net loss from households. These factors substantially reduce the levels of coverage achieved relative to the volume of LLINs financed and delivered, but are not fully captured in current approaches to calculating LLIN needs. Using these inferences, we estimate the levels of coverage achievable with differing volumes of LLIN provision by 2016 under various scenarios of current or modified future levels of distributional efficiency and retention by households.

Despite dramatic gains, current levels of ITN access and use remain well below international targets. Unless inefficiencies due to uneven distribution and rapid net loss can be addressed, meeting international targets will require substantially more nets than currently assumed.

